# Immunoexpression of Ki-67, MCM2, and MCM3 in Ameloblastoma and Ameloblastic Carcinoma and Their Correlations with Clinical and Histopathological Patterns

**DOI:** 10.1155/2015/683087

**Published:** 2015-12-28

**Authors:** Ramón Gil Carreón-Burciaga, Rogelio González-González, Nelly Molina-Frechero, Ronell Bologna-Molina

**Affiliations:** ^1^Research Department, School of Dentistry, Universidad Juárez del Estado de Durango (UJED), 34000 Durango, DGO, Mexico; ^2^Health Care Department, Universidad Autónoma Metropolitana, Xochimilco, 04960 Mexico City, DF, Mexico; ^3^Molecular Pathology Area, School of Dentistry, Universidad de la República (UDELAR), 19200 Montevideo, Uruguay

## Abstract

Cell proliferation assays are performed using antibodies against nuclear proteins associated with DNA replication. These nuclear proteins have gained special interest to predict the biological and clinical behaviors of various tumors. The aim of this study was to analyze the presence of Ki-67 protein and the minichromosome maintenance-2 (MCM2) and maintenance-3 (MCM3) proteins in ameloblastoma.* Materials and Methods*. Cell proliferation marker expression levels were assessed via immunohistochemistry in 111 ameloblastoma cases (72 unicystic ameloblastoma samples, 38 solid/multicystic ameloblastoma samples, and 1 ameloblastic carcinoma). The label index was performed as described previously.* Results.* MCM2 and MCM3 showed higher proliferation indexes in all variants of ameloblastoma compared to the classic marker Ki-67. No correlation between the proliferation index and the clinical and protein expression data was observed.* Conclusion.* The results suggest that clinical features do not directly affect tumor cell proliferation. Moreover, the high levels of cellular proliferation of MCM2 and MCM3 compared with Ki-67 may indicate that MCM2 and MCM3 are more sensitive markers for predicting the growth rate and eventually might be helpful as a tool for predicting aggressive and recurrent behaviors in these tumors.

## 1. Introduction

Ameloblastoma (AM) is one of the most frequent odontogenic tumors. AM is characterized by slow growth, local invasiveness, and high recurrence rates if not removed properly [1, 2]. Frequently, late diagnosis of AM is associated with facial deformities and surgical complications [3, 4]. Recent studies have shown that AM is more prevalent in Asian regions compared to regions of North America and Latin America [5, 6]. In 2005, the World Health Organization (WHO) classified odontogenic tumors into solid/multicystic ameloblastoma (SMA), unicystic ameloblastoma (UA), desmoplastic ameloblastoma (DA), and peripheral ameloblastoma (PA) [7]. The malignant counterpart of this tumor, ameloblastic carcinoma (AC), was classified under the category of odontogenic tumors, dividing them into primary and secondary AC, the latter being a malignant transformation of a preexisting benign AM [7].

The SMA histopathology features consist of islands or strands of odontogenic epithelium within a fibrous stroma. Typically, the basal cells of these islands are columnar, hyperchromatic, and lined up in a palisaded fashion. The central cells may be loosely arranged, resembling stellate reticulum. The AU is a cystic lesion lined by ameloblastomatous epithelium and in AD the stromal component dominates, compressing the odontogenic epithelial components. The PA consists of odontogenic epithelium with the same histomorphological cell types and patterns as seen in SMA but the tumor is in peripheral localization [7].

Cell proliferation is an essential process in all living organisms because of its roles in cell growth and the maintenance of tissue homeostasis [8]. The control of proliferation is completely dysregulated in neoplasms [9]. For this reason, the assessment of cell proliferation activity by immunohistochemistry analysis has become an important tool to provide useful information about the behaviors of several tumors [10]. The Ki-67 antigen is preferentially expressed during the late G1 phase of the cell cycle, whereas quiescent cells (G0 phase) lack Ki-67 expression. Because of its absence in quiescent cells (G0 phase), the Ki-67 protein has been widely used as an important tumor prognostic marker [10].

The minichromosome maintenance (MCM) proteins form a family of molecules that function in DNA synthesis in prokaryotic and eukaryotic cells [11]. In eukaryotic cells, MCM proteins are involved in replication complexes. MCM proteins form a heterohexameric ring of MCM2–MCM7 complexes that act as replicative DNA helicase [11, 12].

Several studies have demonstrated that MCM proteins can be used as proliferation markers to predict the behaviors of diverse neoplasms [11, 13]. Therefore, immunohistochemical detection of MCM can be used to distinguish cells that exhibit aberrant cell proliferation activity.

The functions of MCM are still unclear. The individual roles of each of these proteins in the helicase activity and chromatin organization are yet to be determined [11, 12]. Currently, several studies associate MCM2–MCM7 complex proteins with cell growth assessment [11–18]. MCM2 and MCM3 are part of the MCM2–MCM7 complex and have been studied in a variety of neoplastic tissues as prognostic markers [13, 18, 19].

MCM proteins exhibit low regulation during cell cycle arrest (G0 phase), and they are not detectable by immunohistochemistry. In contrast to Ki-67, MCM is expressed in early G1 phase [14–17]. Because of its expression in early G1 phase, MCM studies are relevant for determining tumor behavior. Together, MCM and Ki-67 assessments are helpful as predictive tools in the prognosis of various neoplasms.

Several related studies have assessed cell proliferation molecules in AM and AC [18–21]. However, different results have been reported, likely due to the histologic features and behaviors of AM.

To date, the expression patterns of MCM2 and MCM3 proteins in AM have not been assessed, so proliferation indexes and their associations with the clinical behavior in AM and AC have not been determined. Ki-67 has been a widely studied protein in AM and AC. Therefore, the present study had two objectives: (1) to elucidate and compare the distribution patterns and proliferation indexes of Ki-67, MCM2, and MCM3 in AM and AC and (2) to correlate the results of the first aim with clinical and histopathological parameters of AM or AC patients.

## 2. Materials and Methods

### 2.1. Tissue Samples

This study was evaluated and accepted by the ethics committee of the Faculty of Dentistry at the Universidad Juarez of Durango State (Folio. EC-FO-UJED-01-14).

One hundred and eleven (72 UA, 38 SMA, and 1 AC) samples were chosen from tissues saturated in paraffin blocks. Tissue samples were collected from the Laboratory of Oral Pathology at the Universidad Juarez del Estado de Durango (Mexico), the Universidad de la República-UDELAR (Uruguay), public and private hospitals in Mexico City (Mexico), and the National Institute of Oncology and Radiobiology in Havana (Cuba). All samples were screened and diagnosed according to the WHO classification of odontogenic tumors. Tumoral tissue was sufficient in all selected samples. Clinical data, such as age, sex, anatomic location, and size of tumors, were available.

### 2.2. Immunohistochemistry

The paraffin blocks were sliced into 4 *μ*m thick sections, and the tissue sections were mounted on silanized slides. Immunohistochemistry staining was performed according to a previously described method [19]. Primary antibodies were used against Ki-67 (Clone MIB-1, Monoclonal Mouse, Anti-Human, Dako Corp., Carpinteria, CA, USA), MCM2 (Clone CRTCT2.1 [1.9H5, IgG1, Cambridge, MA, USA], Monoclonal Mouse Anti-Human, kappa, Abcam Corp, Cambridge, MA, USA), and MCM3 (Clone 101, Monoclonal Mouse Anti-Human, IgG1, Dako Corp., Carpinteria, CA, USA). Cervical lymph node tissue was used as the positive control. For the negative controls, the primary antibody was omitted; all tissue sections that lacked the primary antibody were negative.

### 2.3. Immunohistochemical Analysis

The immunoreactivities of all the markers were assessed via quantitative methods using a modification of the method described by Bologna-Molina et al. [22]. Five photomicrographs of each case of AM were acquired. Evaluations of Ki-67, MCM2, and MCM3 staining were performed using selected fields that were rich in neoplasm ameloblastic cells from SMA and AC and along cystic epithelia and in islands and follicles of mural UA.

**Figure 1:**
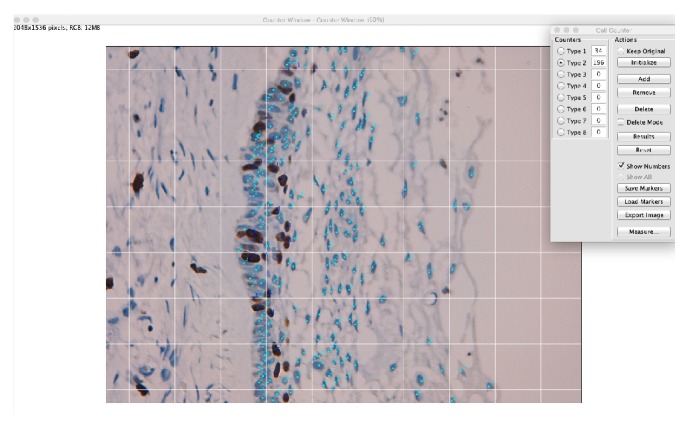
Manual counting technique using ImageJ. The light blue dots indicate negative cells, and dark blue dots indicate positive cells. The upper right quadrant indicates the total positive and negative cells.

It was assumed that Ki-67, MCM2, or MCM3 protein was expressed in the tissue when the nuclei showed a homogeneous, granular, brown, or dark brown staining. The percentage of positive cells was expressed as the ratio between the number of positive tumor cells and the total number of tumor cells counted in the field. Counting was performed manually using an image processing program (ImageJ, http://imagej.nih.gov/ij/) (Figure 1).

### 2.4. Statistical Analysis

Correlations between Ki-67, MCM2, MCM3, and clinical and histopathological parameters were determined using the Spearman correlation coefficient. Cell proliferation indexes (Labeling index, Li) of each marker expressed in tumor epithelium were compared using the Wilcoxon test. This test was also used to compare different types of AM. The results were analyzed using SPSS (version 20.0) statistical software. The results were considered significant if *p* < 0.05.

## 3. Results

### 3.1. Clinical Features of Patients

The median age of the 111 patients with AM was 26 years (mean, 30.5 ± 16.4 years; range, 5–76 years). Sixty men and 51 women were included as patients; therefore, the male : female ratio was 1.2 : 1. A volume increase was registered in all patients. Radiographic medical records of 96 patients were available at the time of the study. Medical records reported one or more AM recurrences in 7 patients, one of them diagnosed as AC. The patient monitoring records were not available for any other patients (Table 1). In most patients (*n* = 96/111, 86.4%), the clinical diagnosis was AM. The remaining patients (*n* = 15/111, 13.5%) were diagnosed as primary intraosseous tumor, intraosseous lesion, or cyst. The median tumor size was 5.0 cm (mean, 5.0 ± 2.24 cm; range, 2–13 cm). Sixty-nine patients (62.1%) were treated with enucleation and curettage; 36 (29.7%) patients, including the AC patient, were treated with en-block mandibular resection or hemimandibulectomy; and six (5.4%) patients were treated with simple curettage.

### 3.2. Immunohistochemical Findings

Nuclear Ki-67, MCM2, and MCM3 immunoexpression levels in AM and AC patients were significantly different. Proliferation index results, evaluated with the Li according to clinical and histopathologic parameters, are described in Tables 1 and 2.

**Figure 2:**
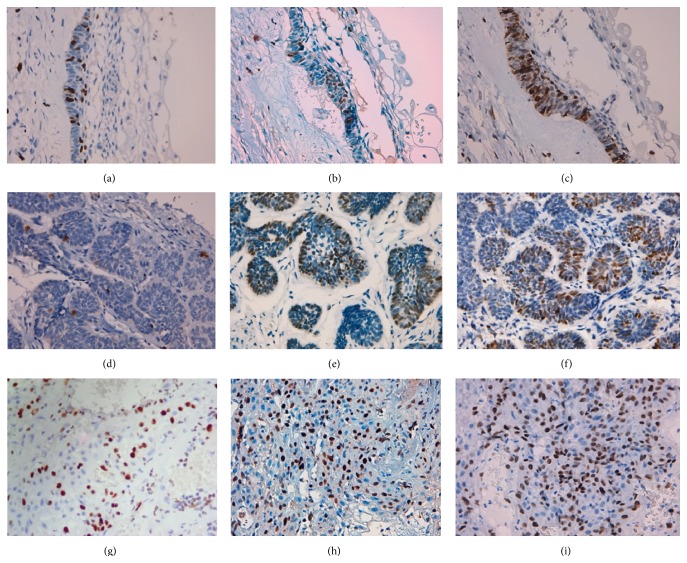
Ki-67, MCM2, and MCM3 immunoexpression in unicystic ameloblastoma with intraluminal proliferation. (a) Ki-67 immunoexpression, 400x, (b) MCM2 immunoexpression, 400x, and (c) MCM3 immunoexpression, 400x. Ki-67, MCM2, and MCM3 immunoexpression in follicular type solid/multicystic ameloblastoma. (d) Ki-67 immunoexpression, 400x, (e) MCM2 immunoexpression, 400x, and (f) MCM3 immunoexpression, 400x. Ki-67, MCM2, and MCM3 immunoexpression in secondary ameloblastic carcinoma. (g) Ki-67 immunoexpression, 400x, (h) MCM2 immunoexpression, 400x, and (i) MCM3 immunoexpression, 400x.

*Ki-67*. Ki-67 expression was weaker compared with MCM2 and MCM3 expression (Table 2, Figures 2(a), 2(d), and 2(g)). The Ki-67 Li was higher in UA compared to SMA (Figures 2(a) and 2(d)); however, the difference was not significant. Ki-67 expression was predominant in higher density areas and in peripheral cells with columnar morphology.

*MCM2*. MCM2 distribution predominated in areas of higher cellular density and in peripheral cells with columnar morphology, while in the central polyhedral cells, expression was minimal or absent (Figures 2(b) and 2(e)). Correlation analysis showed no significant results; however, the proliferation index was higher in SMA (Figures 2(b) and 2(e)).

*MCM3*. MCM3 expression was higher than MCM2 and Ki-67 expression (Table 1, Figures 2(c), 2(f), and 2(i)). Similar to MCM2 and Ki-67, MCM3 expression predominated in areas of higher cell density in peripheral cells with columnar morphology. The MCM3 proliferation index was also higher in SMA, similar to MCM2 (Figures 2(c) and 2(f)).

The Ki-67, MCM2, and MCM3 cellular expression were clearly identified in most of the 110 AM patients, including 104 (94.5%) patients, 108 (98.15%) patients, and 107 (96.4%) patients, respectively.

Only one case of AC was found: a 5 cm tumor in the left mandibular body of a 22-year-old woman. This patient had been previously diagnosed with SMA and had experienced more than one recurrence. The hemimandibulectomy was the only treatment used, followed by monitoring. Histopathological examination reported a secondary AC with tumor-free surgical margins. The last report of the medical record indicates the patient was disease-free. Unfortunately, the medical records of this patient do not include a complete follow-up. For this reason, the patient was reported as lost disease-free. The cell proliferation index was higher for MCM3 compared to MCM2 and Ki-67 (Figures 2(g), 2(h), and 2(i), Table 1).

Table 1 shows that higher proliferation indexes for Ki-67, MCM2, and MCM3 were observed in <26-year-old patients, tumors larger than five centimeters, tumors in the mandibular branch, AM with multilocular radiologic appearance, and nonrecurring tumors.

Spearman correlation analysis and the Wilcoxon test revealed significant (*p* < 0.05) associations between Ki-67, MCM2, and MCM3 and the clinical and histopathologic features (Table 2). However, no significant associations were found between protein expression and the clinical features (Table 1).

## 4. Discussion

Immunohistochemistry is an important tool that can help to determine the biological differences in the behaviors of different tumors [2]. The study of proteins involved in tumor cell proliferation is relevant because these proteins can be used as valuable biomarkers of clinical and biological behavior. Studies of AM have demonstrated that many molecular processes are involved in tumor progression. These processes include proteins involved in cell adhesion [23], bony reconstruction, tumor front [24, 25], apoptosis regulation, cell cycle/cell proliferation [2, 26, 27], and epidermal growth factor gene and BRAF V600 E mutations [28].

Currently, most cell proliferation studies in AM focus on assessing the immunoexpression of proteins involved in this mechanism. The studies associate proteins involved in cell proliferation with histopathologic features, recurrence, and clinical data. Most of these studies report no significant associations between immunoexpression and clinical data [18, 19, 29, 30]. This lack of correlation is consistent with the results of the present research. In our study, proliferation indexes for Ki-67, MCM2, and MCM3 proteins were assessed relative to clinical variables and showed no significant associations. Thus, as proposed by Chae et al. [31], it is possible that there is no clear relationship between proliferative capacity and clinical variables. Although significant associations were not observed in our results, it was found that the highest proliferation indexes were observed in females, in tumors >5 cm, in mandible tumors, in multilocular radiographs, in AC followed by SMA, and in nonrecurring tumors. These results could be related to the study published by Chae et al. [31], which indicated that associated factors such as maxillary location, SMA, UA with mural invasion, AC, and suboptimal treatments are associated with increased growth rate and aggressiveness. It is worth noting that maxillary AM with a higher proliferation index may be related to a greater capacity for AM spread and possible recurrences. McClary et al. [32] suggest that maxillary AM is more aggressive in terms of spread and recurrence because the rate of tumor extension may be associated with the thickness of the cortical maxillary bone, which provides a weak barrier to tumor progression.

Studies of Ki-67 immunoexpression in UA and SMA report varying results [19, 26, 33–35]. In our study, the proliferation index was higher in UA, which could indicate that Ki-67 Li is related to both tumoral behavior [34] and tumoral morphology [19].

In contrast with Ki-67, the immunoexpression levels of MCM2 and MCM3 were higher in SMA. These immunoexpression differences may be related to the cell cycle phase. Ki-67 is expressed from late G1 phase to mitotic (M) phase [17], whereas MCM2 and MCM3 are expressed in the early G1 phase and throughout the cell cycle [17, 36, 37]. It is important to note that MCM family proteins are involved in the early stages of genome replication of eukaryotic cells. MCM proteins are important components of cell replication machinery and ensure that DNA replicates only once per mitotic cycle [38]. Because of this role, the MCM protein family (MCM2 to MCM7) consists of important histological markers to determine cell replication and thus, together with Ki-67, determines prognostic factors in various neoplasms [39].

Studies published by Benevenuto de Andrade et al. [17] in oral nevi and primary melanoma found that MCM2 expression was consistently higher than Ki-67 expression in primary melanoma. This difference suggests that MCM2 is a more sensitive proliferation marker in malignant neoplasms than is Ki-67. These statements are supported by the results of the present study, in which MCM2 expression was higher than Ki-67 expression in AM and AC.

Similarly to MCM2, MCM3 has been studied in different neoplasms. MCM3 may be present in nonproliferating cells, but it signals a readiness to enter the cell cycle. As observed with MCM2, MCM3 protein expression was higher than Ki-67 expression in all AM. This higher MCM3 expression was also described by Lameira et al. [40], who state that MCM3 could be a better marker of proliferation than is Ki-67. Based on these results, it can be suggested that studies with Ki-67, MCM2, and MCM3 may be helpful for monitoring early stages of the cell cycle to identify cells that are in G0 phase with the potential to enter the cell cycle and to identify proliferating cells [39]. Therefore, it is possible to suggest that high expression of MCM2 and MCM3 may be a more reliable marker of proliferation than is Ki-67 in assessing tumor growth and evaluating the potential for recurrence [41].

Studies published by Otero et al. [18], Bologna-Molina et al. [19], and Abdel-Aziz and Amin [20] reported higher proliferative activity in recurrent AM. In the present study, only 6 cases of recurrence were observed. Although no statistical significance was observed, our results were different from those of the above studies. We found that the expression levels of Ki-67, MCM2, and MCM3 were higher in nonrecurring AM. This result is likely affected by the number of patients assessed, the lack of a suitable clinical follow-up, and the surgical technique used, which consisted of conservative surgery in most of our patients. McClary et al. [32] suggested that recurrences due to conservative treatments are the result of persistent slow growth of microscopic disease that takes decades to show up again. Considering the suggestions of McClary et al. and other authors, it is likely that recurrences are associated with surgical treatment [32, 42, 43]. These recurrences might be predicted by high proliferation indexes [18–20]. This prediction can be supported because AC presented the highest proliferation index compared with those of UA and SMA. Although only one case of AC was available, this sample was characterized as an AC arising from SMA with several recurrences due to conservative surgical treatment.

Yoon et al. [44] and Bologna-Molina et al. [45] demonstrated that Ki-67 expression is higher in AC than in AM. This high expression was correlated with AC tumor aggressiveness. Lau et al. [46] found that low MCM2, MCM3, and MCM7 expression levels in medulloblastoma modified cells (cultured in agar) correlated with decreases in the invasion and migration of these cells. MCM2, MCM3, and MCM7 overexpression have been linked to increases in migration, invasion, and proliferation. In our study, Ki-67, MCM2 and MCM3 expression levels were higher in AC than in UA and SMA, which indicate aggressive, invasive, and metastatic neoplasm. Our results may support the conclusion that MCM2 and MCM3 are better markers to evaluate tumoral behavior and more sensitive markers for the identification of proliferating cells than is Ki-67.

As in the case of Ki-67, it is likely that MCM2 and MCM3 expression in AM and AC is not related to the clinical characteristics of these tumors. However, an assessment of MCM2 and MCM3 expression would be useful to predict tumor behavior. Tumors with higher expression indexes might have been associated with greater growth, tumoral invasion, and recurrence. Consistent with other published results [17, 39–41, 46], it is likely that MCM2 and MCM3 expression show more sensitivity for the identification of proliferating cells in AM. Consequently, MCM2 and MCM3 may help to predict tumor behavior. Although there are no available AM studies with MCM2 and MCM3, predicting tumor behavior could be supported by statistical significance between the expression of MCM2 and MCM3 versus Ki-67 in histological subtypes.

The positivity of Ki-67, MCM2, and MCM3 expression in AM and AC is likely the result of cell proliferative activity, while nonproliferating cells that possess the capacity to reenter the cell division cycle are MCM2- and MCM3-positive but Ki-67-negative [39]. This condition would justify the conclusion that different proliferating indexes between Ki-67, MCM2, and MCM3 involve an immunoexpression pattern of proliferating tumor cells positive for Ki-67, MCM2, and MCM3. In contrast, nonproliferating tumor cells subjected to enough stimuli to initiate new proliferation would present an immunoexpression pattern negative for Ki-67 and positive for MCM2 and MCM3. These results are consistent with those of the Endl et al. [39] and Kodani et al. [47] studies, which suggested that MCM2 and MCM3 expression make it possible to distinguish cells with the capacity to reenter cell division and to detect proliferating cells early.

**Figure 3:**
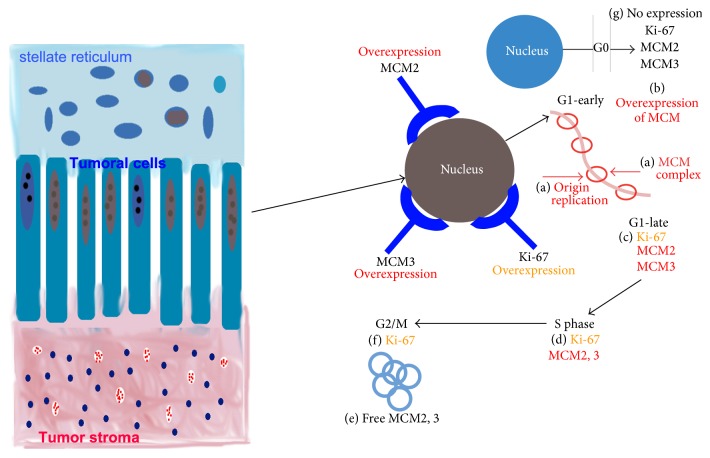
Schematic diagram of possible differences in Ki-67, MCM2, and MCM3 immunoexpression in tumoral cells of ameloblastoma. Unlike Ki-67, the proteins MCM2 and MCM3 are present in G1 early phase. (a) DNA replication origin (licensing) occurs before S phase and in early G1 phase by the loading of minichromosome maintenance protein complexes (MCM2–MCM7). (b) Therefore, the inappropriate expression of MCM2-MCM3 subunits (nuclear overexpression of MCM2 and MCM3) may relate to tumor cells ready to start unregulated proliferation. (c) Ki-67 is overexpressed only in proliferating tumor cells (late G1 phase). Ki-67 could be coexpressed with MCM2-MCM3. (d) During synthesis phase (S phase), Ki-67, MCM2, and MCM3 are in an active replication phase. MCM2-MCM3 complexes are likely free and out of their original complexes during G2/M phases, and they would be ready to start a new replication cycle, (f) while Ki-67 is active. (g) During G0 phase, the MCM2-MCM3 complexes and Ki-67 are inactive; therefore, their expression levels are negative.

Figure 3 explains possible differences in Ki-67, MCM2, and MCM3 protein immunoexpression in AM tumoral cells.

## 5. Conclusion

This study demonstrated that MCM2 and MCM3 immunoexpression were more sensitive than Ki-67 immunoexpression in AM. Additionally, the expression levels of those markers were higher in SMA and AC than in other AM. Therefore, MCM2 and MCM3 may be used as predictive markers of more aggressive tumor behavior and potentially as important markers to predict the risk of malignant transformation.

With regard to AC, due to the rarity of this tumor, this study could only include one case, for which our suggestions and hypotheses must be tested with a larger sample. In this study, no significant correlations between clinical characteristics, recurrence, and protein expression were observed. Thus, we consider a larger number of studies assessing MCM2 and MCM3 in AM and AC with a longer follow-up period and with higher rates of recurrence to be necessary. This study is the first to try to establish a relationship between Ki-67, MCM2, and MCM3 expression and clinical parameters, and the results suggest that there is no direct association between cell proliferation and clinical parameters.

## Figures and Tables

**Table 1:** Li percentages for Ki-67, MCM2, and MCM3 proteins in AM and AC according to clinical and histopathologic parameters.

Factors	*N* = 111/100%	Li%, mean ± SD		
		%Ki-67	%MCM2	%MCM3
Age				
≤26	56/50.4	17.4 ± 13.6	20.3 ± 10.2	34 ± 14.2
>26	55/49.5	13.4 ± 12.8	18.9 ± 10.4	30 ± 15.1
Gender				
Male	60/54	17.3 ± 14.9	18.7 ± 9.4	31.6 ± 30.5
Female	51/45.9	13.1 ± 10.9	20.6 ± 11.3	32.5 ± 15.5
Size				
≤5 cm	75/67.5	14.8 ± 11.9	19.4 ± 9.6	31.8 ± 14.6
>5 cm	36/32.4	16.7 ± 16	19.9 ± 11.8	32.3 ± 15.1
Location				
Maxilla	3/2.7	18 ± 7.3	19.8 ± 2.4	28.5 ± 14.6
Anterior	1/0.9	12.4	18.4	20 ± 2.8
Posterior	2/1.8	20.5 ± 7.7	20.5 ± 3.5	45.4
Mandible	108/97.3	15.1 ± 13.2	19.4 ± 10.2	32.2 ± 14.9
Anterior	9/8.1	16.6 ± 18.4	22.1 ± 12.1	30.6 ± 14.2
Posterior (body and ramus)	99/89.1	15.1 ± 13.2	19.4 ± 10.2	32.2 ± 14.9
Radiography	(*n* = 92/100%)			
Unilocular	(*n* = 64/69.5)	15.5 ± 14.1	19.2 ± 10.6	31.7 ± 15
Multilocular	(*n* = 28/30.4)	16.3 ± 12.1	21.3 ± 11.4	33.2 ± 14.3
Recurrence				
Yes	6/5.4	10.6 ± 4.5	17.3 ± 11.9	23.5 ± 19
No	105/94.5	15.7 ± 13.6	19.7 ± 10.2	32.5 ± 14.4

**Table 2:** Differences in expression among Ki-67, MCM2, and MCM3 proteins according to the Li and histological parameters.

Histologic subtypes	MCM2 versus Ki-67	MCM3 versus Ki-67	MCM2 versus MCM3
UA	***0.000***	***0.011***	***0.000***
SMA	***0.008***	***0.017***	***0.001***
AC	N/A	N/A	N/A

Statistical significance is observed in the histological types of ameloblastoma and Ki -67, MCM2, and MCM3. The bold letter indicates statistical significance (*p* < 0.05), *Spearman* test.
